# Outcomes of WHO-conforming, longer, all-oral multidrug-resistant TB regimens and analysis implications

**DOI:** 10.5588/ijtld.22.0613

**Published:** 2023-06-01

**Authors:** M. L. Rich, U. Khan, C. Zeng, A. LaHood, M. F. Franke, S. Atwood, M. Bastard, E. Burhan, N. Danielyan, P. M. Dzhazibekova, D. Gadissa, A. Ghafoor, C. Hewison, M. S. Islam, E. Kazmi, P. Y. Khan, L. Lecca, L. B. Maama, N. Melikyan, Y. Y. Naing, K. Philippe, N. A. Saki, K. J. Seung, A. Skrahina, G. B. Tefera, F. Varaine, S. C. Vilbrun, L. Võ, C. D. Mitnick, H. Huerga

**Affiliations:** 1 Division of Global Health Equity, Brigham and Women’s Hospital, Boston, MA; 2 Partners In Health, Boston, MA, USA; 3 Interactive Research & Development Global, Singapore, Singapore; 4 Department of Global Health and Social Medicine, Harvard Medical School, Boston, MA, USA; 5 Epicentre, Paris, France; 6 Persahabatan General Hospital, Jakarta, Indonesia; 7 Médecins Sans Frontières (MSF), Tbilisi, Georgia; 8 National Science Center, Almaty, Republic of Kazakhstan; 9 Partners In Health (PIH), Addis Ababa, Ethiopia; 10 National Tuberculosis Programme (NTP), Ministry of National Health, Islamabad, Pakistan; 11 MSF, Paris, France; 12 Interactive Research & Development, Dhaka, Bangladesh; 13 Directorate General Health Services, Centers for Disease Control and Prevention, Sindh, Pakistan; 14 Department of Clinical Research, London School of Hygiene & Tropical Medicine, London, UK; 15 Socios En Salud Sucursal, Lima, Peru; 16 PIH, Maseru, Lesotho; 17 NTP, Maseru, Lesotho; 18 MSF, Yerevan, Armenia; 19 MSF, Yangon, Myanmar; 20 Zanmi Lasante, Cange, Haiti; 21 World Health Organization, Country Office, Dhaka, Bangladesh; 22 MSF, Minsk, Belarus; 23 GHESKIO Institute of Infectious Diseases and Reproductive Health, NTP, Port-au-Prince, Haiti; 24 Friends for International TB Relief, Ho Chi Minh City, Vietnam

**Keywords:** effectiveness, MDR-TB, RR-TB, fluoroquinolone, rifampicin resistance, multidrug-resistant TB

## Abstract

**BACKGROUND:**

Evidence of the effectiveness of the WHO-recommended design of longer individualized regimens for multidrug- or rifampicin-resistant TB (MDR/RR-TB) is limited.

**OBJECTIVES:**

To report end-of-treatment outcomes for MDR/RR-TB patients from a 2015–2018 multi-country cohort that received a regimen consistent with current 2022 WHO updated recommendations and describe the complexities of comparing regimens.

**METHODS:**

We analyzed a subset of participants from the endTB Observational Study who initiated a longer MDR/RR-TB regimen that was consistent with subsequent 2022 WHO guidance on regimen design for longer treatments. We excluded individuals who received an injectable agent or who received fewer than four likely effective drugs.

**RESULTS:**

Of the 759 participants analyzed, 607 (80.0%, 95% CI 77.0–82.7) experienced successful end-of-treatment outcomes. The frequency of success was high across groups, whether stratified on number of Group A drugs or fluoroquinolone resistance, and ranged from 72.1% to 90.0%. Regimens were highly variable regarding composition and the duration of individual drugs.

**CONCLUSIONS:**

Longer, all-oral, individualized regimens that were consistent with 2022 WHO guidance on regimen design had high frequencies of treatment success. Heterogeneous regimen compositions and drug durations precluded meaningful comparisons. Future research should examine which combinations of drugs maximize safety/tolerability and effectiveness.

## Abstract (fr)

**CONTEXTE:**

Les données probantes portant sur l’efficacité de schémas thérapeutiques individualisés plus longs recommandés par l’OMS pour la TB multirésistante ou résistante à la rifampicine (MDR/RR-TB) sont limitées.

**OBJECTIFS:**

Rapporter les résultats de fin de traitement des patients atteints de MDR/RR-TB, issus d’une cohorte multi-pays de 2015–2018, ayant reçu un schéma thérapeutique conforme aux recommandations actuelles de l’OMS mises à jour en 2022 et décrire les complexités de la comparaison des schémas thérapeutiques.

**MÉTHODES:**

Nous avons analysé un sous-ensemble de participants à l’étude observationnelle endTB qui ont commencé un schéma thérapeutique plus long contre la MDR/RR-TB, conforme aux directives de l’OMS de 2022 sur les schémas de traitements plus longs. Nous avons exclu les personnes ayant reçu un agent injectable ou ayant reçu moins de quatre médicaments susceptibles d’être efficaces.

**RÉSULTATS:**

Sur les 759 participants analysés, 607 (80,0% ; IC 95% 77,0–82,7) ont obtenu des résultats positifs en fin de traitement. La fréquence de traitements réussis était élevée dans tous les groupes, qu’ils soient stratifiés sur le nombre de médicaments du groupe A ou sur la résistance aux fluoroquinolones, et se situait entre 72,1% et 90,0%. Les schémas thérapeutiques étaient très variables en ce qui concerne la composition et la durée de prise des différents médicaments.

**CONCLUSIONS:**

Les traitements plus longs, entièrement oraux, individualisés et conformes aux recommandations 2022 de l’OMS pour les schémas thérapeutiques ont été associés à une fréquence élevée de réussite thérapeutique. L’hétérogénéité des compositions des schémas thérapeutiques et des durées d’administration des médicaments n’a permis aucune comparaison significative. Les recherches futures devraient examiner quelles associations de médicaments maximisent l’innocuité/la tolérance et l’efficacité.

Multidrug- or rifampicin-resistant TB (MDR/RR-TB) newly afflicts more than 500,000 people annually and has a global treatment success rate of 60%.^1^ In 2019, the WHO recommended a major change to the drug hierarchy for the stepwise design of longer individualized treatment regimens for MDR/RR-TB.^2^ By deprioritizing injectable agents to the third tier, the drug hierarchy effectively recommended all-oral regimens for all forms of MDR/RR-TB, including fluoroquinolone (FQ) resistant MDR/RR-TB,^3^–^5^ and represented a significant departure from prior guidelines in which the relatively toxic injectable agents (an aminoglycoside or capreomycin) were a cornerstone of treatment.

The 2019 revisions to WHO treatment recommendations^2^ were derived primarily from findings from an individualized patient data (IPD) meta-analysis of data from observational cohort studies and a few randomized controlled trials.^6^ Analyses focused on estimating the effectiveness of individual drugs used for at least 1 month, rather than the effectiveness of combinations of drugs. For example, assignment of late-generation FQs to Group A was based on analyses comparing outcomes among patients receiving a regimen-containing moxifloxacin or levofloxacin for ≥1 month at any point during treatment, to those from regimens that never contained either (or contained them for <1 month).^2^ The resulting recommendations were “conditional”, reflecting “very low certainty” evidence. The WHO acknowledged that longer-regimen recommendations (for example, constructing a regimen with three Group A plus at least one Group B drug when no Group A or B drug resistance exists) had not been tested in either research or programmatic conditions. The 2022 Guidelines update retains this drug hierarchy.^5^

The most recent update recommends three different short all-oral regimens^5^,^7^ while retaining the option of the longer, individualized regimen in some circumstances. Consequently, evidence on the effectiveness and optimization of longer regimens constructed according to WHO hierarchy remain critically important.

Here, we report end-of-treatment outcomes for patients from a large multi-country 2015–2018 cohort who received an individualized, all-oral longer MDR/RR-TB regimen consistent with both 2019 and 2022 WHO recommendations.^2^,^5^ We report the heterogeneity in composition and duration of the regimens, even among those that conform to WHO guidelines, and highlight implications for analyses.

## METHODS

We conducted an analysis of data from the endTB Observational Study (Clinicaltrials.gov identifier: NCT03259269), implemented by national TB programmes (NTPs) with support from endTB consortium partners.^8^ The observational study comprised a prospective cohort of patients receiving individualized longer treatments for MDR/RR-TB, containing bedaquiline (BDQ) and/or delamanid (DLM), in one of 17 participating countries.^8^–^10^

### Study population

We included patients who received a first endTB treatment regimen for MDR/RR-TB between 1 April 2015 and 30 September 2018, provided informed consent for inclusion in the observational study, had documented rifampicin resistance and received regimens equivalent to what would be recommended in the latest 2022 WHO guidance.^5^ Because the focus of this report was all-oral, WHO-conforming treatment regimens, we excluded individuals initiating an injectable agent, including a carbapenem, at any point during treatment, as well as patients who received fewer than four effective drugs.

### Data collection

Collection of routinely captured clinical and laboratory data was organized by the three endTB consortium partners and standardized across all sites.^11^ Data were entered into a common Electronic Medical Record (EMR) system.^12^,^13^

### Regimen design and definitions

Longer (18–21 months) individualized treatment regimens were constructed by the local treating physician according to NTP guidelines and WHO 2016 Guidelines,^14^ and informed by the endTB clinical guide.^15^ We defined treatment as consistent with 2022 WHO guidance^5^ if the baseline regimen was all-oral and contained at least four likely effective drugs, and if it never included an injectable agent (i.e., aminoglycosides, capreomycin, carbapenems). A drug was deemed likely effective based on existence of at least one of the following: confirmed susceptibility of the strain infecting the individual patient and/or no previous use of the medicine for >1 month.

### Outcome

We calculated treatment outcomes based on the WHO framework for outcome definitions.^16^ While treatments ≤15 months were not anticipated, they occurred in 32 (4.2%) of 759 patients. Since longer regimens are intended to last 18–21 months, we imposed a minimum treatment duration of 15 months for evaluation of treatment completion and cure.^8^ Patients without indication of loss to follow-up or death who were treated for <15 months were assigned an outcome as follows: 1) an outcome was classified as “treatment failure” if the patient had at least two culture results after 8 months and at least one of the following was true: more than one of the last three cultures were positive or the last culture was positive; 2) an outcome of “<15 months, favorable” was assigned if patient had experienced culture conversion and had no subsequent positive cultures; 3) an outcome was classified as “<15 months, unfavorable” if there were fewer than two cultures after 8 months of treatment and there was no evidence of culture conversion.^17^

### Statistical analyses

We report the number and frequency of end-of-treatment outcomes across groups defined by the baseline FQ resistance and number of WHO Group A, B and C drugs in the initial baseline regimen. We also report the distribution (median, 25^th^ and 75^th^ percentiles) of other key regimen characteristics, including the duration of the baseline regimen, the duration of each Group A drug included in the baseline regimen, and the number of drugs in the regimen likely and not likely to be effective. Analyses were conducted using SAS v9.4 (SAS Institute, Cary, NC, USA) and RStudio, PBC v1.4.1106 (R Computing, Vienna, Austria).

### Research ethics

The endTB Observational Study protocol received local ethical approval in all endTB countries, as well as central ethics review committees for each of the three partners (Partners Human Research Committee, Médecins Sans Frontiers Ethics Review Board, and Interactive Research and Development Institutional Review Board). Participants provided written informed consent for inclusion in the observational cohort.

## RESULTS

### Overview of the study cohort

A total of 2,789 patients consented to participate in the endTB observational study and initiated a BDQ-and/or DLM-containing regimen for the treatment of MDR/RR-TB. Of these, 759 met the inclusion criteria for these analyses. The median age at treatment initiation was 36.5 years (interquartile range [IQR] 28–49); 288 (37.9%) were women (Table 1). Comorbidities were common: respectively 21.7% (164/755), 21.5% (157/729) and 4.2% (31/746) were living with HIV, diabetes mellitus and hepatitis C (as defined by a positive antibody test). FQ drug susceptibility testing (DST) was susceptible in 353 (46.5%) and resistant in 219 (28.9%); DST not done or unknown in 187 (24.9%). Furthermore, 324/614 (52.8%) of patients had cavitary TB on chest X-ray and 338/759 (44.5%) had received prior treatment with second-line TB drugs (Table 1, Supplementary Table S1).

**Table 1:** Characteristics of patients receiving an all-oral regimen conforming to 2020 WHO guidance for RR/MDR-TB (n = 759) ^*^

Characteristic	*n* (%)^*^
Demographic	
Age at treatment initiation, years, median [IQR]	36.5 [28–49]
Female	288 (37.9)
Comorbidities	
DM or glucose intolerance (*n* = 729)^†^	157 (21.5)
HIV infection (*n* = 755)	164 (21.7)
Hepatitis B virus infection (*n* = 746)^‡^	49 (6.6)
Hepatitis C virus infection (*n* = 745)^§^	31 (4.2)
At least one comorbidity other than those above	67 (8.8)
TB-related	
Prior TB treatment with second-line drugs	338 (44.5)
Bilateral disease (*n* = 632)^¶^	427 (67.6)
Cavitary disease (*n* = 614)^¶^	324 (52.8)
Smear-positive sputum (*n* = 658)	309 (47.0)
Cavitary disease and smear status (*n* = 547)	
No cavitary disease, smear <3+	226 (29.8)
Cavitary disease, smear <3+	262 (34.5)
No cavitary disease, smear 3+	22 (2.9)
Cavitary disease, smear 3+	37 (4.9)
Resistance profile	
RR/MDR-TB with FQ susceptibility	353 (46.5)
RR/MDR-TB with FQ resistance	219 (28.9)
RR/MDR-TB with FQ DST unknown	187 (24.6)
Body mass index <18.5 (*n* = 741)	330 (44.5)

^*^ Unless otherwise noted, *n* = 759.

^†^ For the purposes of assessing DM disease control, we considered HbA1c results taken up to 90 days before initiation of the BDQ- or DLM-containing regimen or up to 15 days after, with preference given to before initiation.

^‡^ Hepatitis B virus surface antigen-positive.

^§^ Hepatitis C virus antibody-positive.

^¶^ Baseline chest radiograph was defined as the Xray taken before initiation of the BDQ- or DLM-containing regimen or up to 15 days after, with preference given to before initiation.

RR/MDR-TB = rifampin/multidrug-resistant TB; IQR = interquartile range; DM = diabetes mellitus; FQ = fluoroquinolone; DST = drug susceptibility testing; HbA1c = glycated hemoglobin; BDQ = bedaquiline; DLM = delamanid.

### End-of-treatment outcomes

Overall, 80% (607/759) patients experienced a successful end-of-treatment outcome. Stratified by FQ DST results, treatment success was recorded in 77.6% (274/353) patients with FQ susceptibility, 85.8% (188/219) with FQ resistance and 77.7% (145/187) with unknown FQ susceptibility (Table 2, Supplementary Table S2). The cohort of patients with FQ DST results was further stratified by the number of WHO Group A, B and C drugs, with the frequency of successful end-of treatment outcomes, ranging from 72.1% to 90.0% (Table 3, Supplementary Table S3).

**Table 2:** Frequency of end-of-treatment outcomes among patients receiving an all-oral regimen conforming to 2020 WHO guidance for rifampicin-resistant or multidrug-resistant TB, stratified by baseline FQ resistance (n = 759)

FQ resistance	*n* (%)	End-of-treatment outcome					

		Favorable^*^	Unfavorable			Not evaluated
		
		*n* (%) (95% CI)	Failure^†^ *n* (%)	Death *n* (%)	LTFU or no data *n* (%)	*n* (%)
Any	759 (100.0)	607 (80.0) (77.0–82.7)	13 (1.7)	88 (11.6)	45 (5.9)	6 (0.8)
Susceptible	353 (46.5)	274 (77.6) (73.0–81.7)	7 (2.0)	42 (11.9)	28 (7.9)	2 (0.6)
Resistant	219 (28.9)	188 (85.8) (80.6–89.9)	5 (2.3)	13 (5.9)	12 (5.5)	1 (0.5)
Unknown	187 (24.6)	145 (77.5) (71.0–83.0)	1 (0.5)	33 (17.7)	5 (2.7)	3 (1.6)

^*^ Includes outcomes of cured, completed, and “< 15 months, favorable”.

^†^ Includes outcomes of “< 15 months, unfavorable”.

FQ = fluoroquinolone; CI = confidence interval; LTFU = loss to follow-up.

**Table 3:** Frequency of end-of-treatment outcomes among patients receiving an all-oral regimen conforming to 2020 WHO guidance for rifampin-resistant or multidrug-resistant TB, stratified by number of Group A, B and C drugs in the baseline regimen and FQ resistance (n = 572) ^*^

Number of likely effective Group A drugs	Number of likely effective Group B and C drugs^†^	FQ resistance	*n*	End-of-treatment outcome					

				Favorable		Unfavorable			Not evaluated
		
				*n* (%)	(95% CI)	Failure *n* (%)	Death *n* (%)	LTFU or no data *n* (%)	*n* (%)
Exactly 3	1 Group B + 0 Group C	FQ-S	65	55 (84.6)	(73.8–91.6)	1 (1.5)	4 (6.2)	5 (7.7)	0 (0.0)
	≥1 Group B + ≥0 Group C^‡^		71	59 (83.1)	(72.6–90.2)	0 (0.0)	8 (11.3)	4 (5.6)	0 (0.0)
	0 Group B + ≥1 Group C		10	9 (90.0)	(57.4–100.0)	1 (10.0)	0 (0.0)	0 (0.0)	0 (0.0)
Exactly 2	≥2 Group B and/or Group C	FQ-S	139	102 (73.4)	(65.5–80.1)	4 (2.9)	16 (11.5)	17 (12.2)	0 (0.0)
Exactly 2^§^	≥2 Group B and/or Group C	FQ-R	184	162 (88.0)	(82.5–92.0)	4 (2.2)	10 (5.4)	7 (3.8)	1 (0.5)
Exactly 1	≥3 Group B and/or Group C	FQ-S	68	49 (72.1)	(60.4–81.4)	1 (1.5)	14 (20.6)	2 (2.9)	2 (2.9)
Exactly 1	≥3 Group B and/or Group C	FQ-R	35	26 (74.3)	(57.8–86.0)	1 (2.9)	3 (8.6)	5 (14.3)	0 (0.0)

^*^ 187 patients were excluded from this analysis because they did not have FQ susceptibility testing results.

^†^ All groups are mutually exclusive.

^‡^ Patients in this group had at least 5 likely effective drugs.

§ 100% of the group A drugs were BDQ and LZD.

FQ=fluoroquinolone; CI=confidence interval; LTFU=loss to follow-up; FQ-S=fluoroquinolone-susceptible; FQ-R=fluoroquinolone-resistant; BDQ=bedaquiline; LZD = linezolid.

### Heterogeneity of regimens

While all patients received a WHO-conforming regimen, there was considerable variability in the composition of these regimens in terms of the number of unique regimens used within a category. For example, among individuals who initiated treatment with three Group A, at least one Group B and any number of Group C drugs, there are 24 regimens with unique drug combinations (Table 4). Regimens also varied in the number of likely non-effective drugs included in the regimen (typically containing between zero and two) and the duration of treatment with BDQ and linezolid (Table 4). For patients with FQ susceptibility, the median duration of BDQ was around 6 months, with the majority of patients receiving the drug for less than a year. This contrasts with the BDQ durations observed among patients with FQ resistance, of whom around 25% were treated with BDQ for ≥20 months. In most patients (80–90%), the baseline regimen was not maintained for the entire duration of treatment, with the median time to baseline change occurring between 2.9 and 5.6 months (median, 25^th^ and 75^th^ percentiles) after treatment initiation.

**Table 4:** Characteristics of all-oral regimens conforming to 2020 WHO guidance for rifampin-resistant or multidrug-resistant TB, stratified by number of Group A, B and C drugs in the baseline regimen and FQ resistance (n = 572) ^*^

**A)**			
Drug groups	Number of Group A drugs: exactly 3		

	1 Group B + 0 Group C FQ-S (*n* = 65) median [IQR]	≥1 Group B + ≥0 Group C FQ-S (*n* = 71) median [IQR]	0 Group B + ≥1 Group C FQ-S (*n* = 10) median [IQR]
Number of unique initial regimens	2	24	7
Number of likely effective drugs in initial regimen	4 [4–4]	5 [5–6]	4 [4–5]
Number of unlikely effective drugs in initial regimen	1 [1–2]	1 [0–1]	1 [1–1]
Duration of initial regimen, months^†^	5.6 [3.5–8.3] (*n* = 65)	4.8 [1.3–5.6] (*n* = 71)	5.5 [1.2–6.6] (*n* = 10)
Duration of LZD administration,^‡^ months	19.7 [15.5–20.0] (*n* = 65)	19.4 [10.0–21.0] (*n* = 71)	14.3 [9.1–21.3] (*n* = 10)
Duration of BDQ administration,^‡^ months	6.4 [5.5–10.9] (*n* = 65)	5.6 [5.5–9.3] (*n* = 71)	8.2 [5.7–9.1] (*n* = 10)
Duration of LFX/MFX administration,^‡^ months	19.7 [9.0–20.0]	19.4 [16.4–21.6]	19.1 [9.1–21.3]
Ever had regimen change, *n* (%)	58 (89)	63 (89)	9 (90)
Duration of initial regimen among those with a regimen change, months	5.6 [3.6–8.3]	5 [1.7–5.6]	5.5 [1.2–5.7]
Duration of treatment,^§^ months	20 [18.3–20.3]	20 [18.3–22.2]	19.3 [9.3–21.5]

**B)**				
Drug groups	Number of Group A drugs			

	Exactly 2 Group A drugs		Exactly 1 Group A drug	
	
	Group B and/or Group C (*n* = 139) FQ-S median [25^th^–75^th^]	Group B and/or Group C (*n* = 184) FQ-R median [25^th^–75^th^]	Group B and/or Group C (*n* = 68) FQ-S median [25^th^–75^th^]	Group B and/or Group C (*n* = 35) FQ-R median [25^th^–75^th^]
Number of unique initial regimens	48	42	19	27
Number of likely effective drugs in initial regimen	5 [4–5]	5 [4–5]	5 [5–5]	5 [4–5]
Number of unlikely effective drugs in initial regimen	1 [0–1]	1 [1–2]	1 [0–1]	1 [1–2]
Duration initial regimen, months^†^	4.5 [1.2–6.6] (*n* = 10)	5.6 [4.0–9.4] (*n* = 184)	2.4 [0.5–5.6] (*n* = 14)	5.2 [1.4–6.2] (*n* = 31)
Duration of LZD administration,^‡^ months	18.4 [9.1–21.3] (*n* = 10)	20 [18.8–24.0] (*n* = 184)	14.5 [4.3–19.7] (*n* = 7)	18.6 [9.1–22.7] (*n* = 15)
Duration of BDQ administration,^‡^ months	6.1 [5.7–9.1] (*n* = 10)	11.7 [6.0–20.0] (*n* = 41)	5.5 [2.2–14.7] (*n* = 68)	16.4 [6.0–19.7] (*n* = 21)
Duration of LFX/MFX administration,^‡^ months	19.2 [9.1–21.3]	20 [13.2–24.0]	18.4 [11.5–19.9]	18.3 [1.8–21.6]
Ever had regimen change, *n* (%)	116 (83)	162 (88)	61 (90)	28 (80)
Duration of initial regimen among those with a regimen change, months	4.4 [1.2–5.7]	5.6 [3.7–8.9]	2.9 [0.5–5.7]	4.7 [1.2–6.0]
Duration of treatment,^§^ months	20 [9.3–21.5]	20.9 [20.0–24.3]	19.8 [13.2–20.7]	20.9[10.7–24.3]

^*^ 187 patients were excluded from this analysis because they did not have FQ susceptibility testing results.

^†^ Duration of initial regimen represents the minimum of time to first regimen change or to end of treatment outcome.

^‡^ Duration of drug counted from Day 2 of treatment; prescribed interruptions did not count toward duration.

^§^ Number of months between the treatment start date and the end of treatment; includes any prescribed interruptions. FQ = fluoroquinolone; FQ-S = FQ-susceptible; LZD = linezolid; BDQ = bedaquiline; LFX = levofloxacin; MFX = moxifloxacin; FQ-R = FQ-resistant.

## DISCUSSION

This report provides evidence on the performance of longer, all-oral treatment regimens conforming to 2019 and 2022 WHO DR-TB Guidelines.^2^,^5^ All-oral WHO-conforming longer regimens were effective, with a frequency of treatment success of 80.0% (95% confidence interval [CI] 77.0–82.7) as compared to the historical global average of 59%.^1^ Furthermore, multiple combinations of Group A, B and C drugs resulted in a high frequency of favorable end-of-treatment outcomes. These results also compare well to those from key trials, including STREAM (Evaluation of a Standardised Treatment Regimen of Anti-tuberculosis Drugs for Patients with Multidrug-resistant Tuberculosis) stage II^18^ (comparing different shorter regimens) and TB-PRACTECAL^19^ (comparing shorter all-oral and longer regimens) for MDR/RR-TB, and to those from the single arm Nix-TB trial (6 months of BDQ-pretomanid-linezolid) for FQ-resistant MDR/RR-TB.^20^ They also compare favorably with the WHO-recommended, all-oral shorter regimens being used today.^5^ While shorter regimens are not constructed based on a stepwise individualized approach, most prioritize Group A drugs.

Due to the variability in regimen composition within categories, we chose not to directly compare the frequency of favorable outcomes for regimens having different numbers of Group A drugs. Therefore, the frequencies reported here do not account for differences in patient characteristics, such as disease severity, prior treatment with first- and second-line drugs, and comorbidities, such as HIV. While such comparisons are desirable, our findings regarding the heterogeneous nature of treatment highlight the complexities of comparative effectiveness analyses of longer individualized regimens for MDR/RR-TB. First, nearly all (80–90%) of patients had a change in their baseline regimen. Because regimens change over time, analyses that use baseline regimen classifications to estimate an intention-to-treat effect will not always adequately reflect relevant drug exposure throughout the course of treatment, or capture the reasons driving these changes, data that are essential to understanding regimen effectiveness.^21^,^22^ Second, our results show that patients with similar regimen compositions often received different durations of key drugs, such as BDQ and linezolid. These varying durations were driven by tolerability/toxicity, as well as variability in local norms, treatment response and/or regimen strength. Comparative analyses that fail to account for heterogeneous drug durations implicitly treat all durations as contributing equally to regimen effectiveness (i.e., they discount the potential for a duration response), which can obscure treatment effects.

A third challenge to comparative effectiveness analyses of longer regimens is the potential for heterogeneity in drug effectiveness within WHO groupings (i.e., drugs within the same group do not necessarily make the same contributions to the regimen). Variability in contribution may be due to differences in anti-TB activity, lack of safety or tolerability, or interactions with other drugs in the regimen. Because regimens composed of the same number of drugs per group may have varying effectiveness, the most informative comparisons may be those that compare specific regimens rather than those characterized by the number of drugs per group. The WHO 2022 Guidelines take a step in this direction with multiple direct regimen comparisons.^5^ When large longitudinal datasets are available, application of the target trial framework, along with statistical methods (e.g., inverse probability weighting, *g*-formula) that enable study of time-varying treatments, can address many of these challenges,^21^,^23^ and may facilitate the generation of evidence to inform treatment guidance with a higher degree of certainty.

Limitations of the present analyses relate to the application of the 2019/2022 Guidelines to a cohort of patients whose treatment preceded these guidelines. First, the regimens were designed by clinicians in accordance with different guidance^14^,^15^ than the one being used to select for inclusion in the present analysis. Regimens included here conform to both sets of guidance, but they are not optimized according to the 2019/2022 WHO Guidelines (e.g., patients who received one Group A drug in treatment that started in 2017 might have received three if treatment had started in 2021). As a result, the results presented here may underestimate treatment success compared to those that will occur in a similar cohort in which treatment is designed in accordance with the 2019/2022 WHO Guidelines. Second, patients who did receive a regimen consistent with subsequent WHO Guidelines may have differed in important ways from those who did not. For example, individuals who received fewer than four likely effective drugs (and were excluded from the present analysis) could be more likely to have had previous treatment with second-line drugs and/or extensive resistance. The frequency of favorable outcomes in our cohort could, consequently, represent an overestimate relative to the frequency among all-comers in the future. Classification of likely-effectiveness is imperfect,^24^,^25^ and it is possible that drugs classified as not likely-effective did in fact contribute to regimen effectiveness. It is conceivable that the encouraging results presented here discourage the practice of adding extra drugs during prospective application of the 2019/2022 guidance. The result could be reduced effectiveness relative to that reported in the present cohort. Despite these limitations, these findings offer high-level insight into the effectiveness of longer regimens constructed according to 2019/2022 WHO guidance.

## CONCLUSION

Treatment regimens consistent in design with the WHO stepwise algorithm for MDR/RR-TB can achieve a frequency of treatment success surpassing 80%, when two or more Group A drugs are used in a four-drug regimen. Additional research is needed to determine the optimal drug combinations. Because the number of clinical trials with direct regimen comparisons is extremely limited, observational data will continue to play a key role in identifying the optimal treatment for MDR/RR-TB.^22^ The application of approaches that facilitate improved inference from observational cohorts of patients receiving shorter and longer regimens implemented under routine programmatic conditions will be of critical importance to generating high-quality evidence for MDR/RR-TB treatment, with the overall goal of reducing TB morbidity and mortality and improving patient quality of life.
